# Identification of Chimeric Repressors that Confer Salt and Osmotic Stress Tolerance in *Arabidopsis*

**DOI:** 10.3390/plants2040769

**Published:** 2013-12-05

**Authors:** Daisuke Kazama, Masateru Itakura, Takamitsu Kurusu, Nobutaka Mitsuda, Masaru Ohme-Takagi, Yuichi Tada

**Affiliations:** 1 Graduate School of Bionics, Tokyo University of Technology, 1404-1 Katakura, Hachioji, Tokyo 192-0982, Japan; E-Mail: b010709749@bss.teu.ac.jp; 2 School of Bioscience and Biotechnology, Tokyo University of Technology, 1404-1 Katakura, Hachioji, Tokyo 192-0982, Japan; E-Mails: teru.utau@gmail.com (M.I.); kurusutkmt@stf.teu.ac.jp (T.K.); 3 Bioproduction Research Institute, National Institute of Advanced Industrial Science and Technology (AIST), Tsukuba, Ibaraki 305-0053, Japan; E-Mails: Nobutaka.Mitsuda@aist.go.jp (N.M.); m-takagi@aist.go.jp (M.O.-T.); 4 Institute for Environmental Science and Technology, Saitama University, Saitama-shi, Saitama 338-8570, Japan

**Keywords:** CRES-T, osmotic stress, repressor, salt stress, stress tolerance, transcription factors

## Abstract

We produced transgenic *Arabidopsis plants* that express chimeric genes for transcription factors converted to dominant repressors, using Chimeric REpressor gene-Silencing Technology (CRES-T), and evaluated the salt tolerance of each line. The seeds of the CRES-T lines for ADA2b, Msantd, DDF1, DREB26, AtGeBP, and ATHB23 exhibited higher germination rates than Wild type (WT) and developed rosette plants under up to 200 mM NaCl or 400 mM mannitol. WT plants did not grow under these conditions. In these CRES-T lines, the expression patterns of stress-related genes such as *RD29A*, *RD22*, *DREB1A*, and *P5CS* differed from those in WT plants, suggesting the involvement of the six transcription factors identified here in the stress response pathways regulated by the products of these stress-related genes. Our results demonstrate additional proof that CRES-T is a superior tool for revealing the function of transcription factors.

## 1. Introduction

Salinity stress is one of the most significant factors limiting the productivity of agricultural crops. Hence, improving salt tolerance in crops is essential for sustainable food production. To identify key genes that are involved in plant salt tolerance, numerous candidate genes for salt tolerance have been overexpressed in plants and their effects on salt tolerance have been evaluated. For example, overexpression of a vacuolar Na^+^/H^+^ antiporter from *Arabidopsis thaliana* in *Arabidopsis* plants was shown to promote sustained growth and development in soil watered with up to 200 mM sodium chloride [1]. Other salt tolerance genes include the genes for late-embryogenesis abundant proteins from barley [2], vacuolar H^+^-pyrophosphatase from *A. thaliana* [3], *SALT OVERLY SENSITIVE 1* [4] from *A. thaliana*, and various transcription factors. Transcription factors play a pivotal role in developing stress tolerance in plants against various environmental stresses. Overexpression of more than 40 transcription factors has been reported to confer enhanced salt tolerance in transgenic plants [5]. For example, ectopic expression of *DREB1A*, that encodes an AP2/ERF (APETALA2/Ethylene-Responsive Element Binding Factor), activates the expression of certain stress-inducible genes in *Arabidopsis*, with resultant improved tolerance of drought, salt, and freezing [6]. Expression of the C2H2-type zinc finger protein ZFP182 from rice increased tolerance to salt stress in both transgenic tobacco and rice [7]. Transgenic rice plants over-expressing rice SNAC1 (stress-responsive NAC1) and SNAC2 showed significantly higher drought and salinity stress tolerance than wild-type (WT) [8,9]. Ectopic expression of AtMYB44 enhances drought and salt tolerance in *Arabidopsis* [10]. A number of *Arabidopsis* transcription factors involved in salt tolerance were identified via the mini-scale Full-length cDNA Over-eXpressor (FOX) gene hunting system [11].

In addition, loss-of-function mutants of transcription factors CBF2/DREB1C have elevated tolerance to abiotic stresses [12]; however, there have been few reports on the effects of suppressing transcription factors on plant abiotic stress tolerance. One of the reasons for the lack of such studies is that plants possess functionally redundant transcription factors and suppression of one transcription factor does not necessarily result in a loss-of-function phenotype. Recently, Chimeric REpressor gene-Silencing Technology (CRES-T) has been developed to suppress expression of plant transcription factors [13]. In CRES-T, a transcription factor is converted to a strong repressor by fusion with the EAR-motif repression domain SRDX [14] and dominantly suppresses the expression of target genes to generate loss-of-function-type mutant plants, even in the presence of functionally redundant transcriptional activators [13]. Thus, CRES-T has a great advantage over other sequence-dependent gene silencing procedures such as antisense RNA and RNAi (RNA interference). Using CRES-T, Mito *et al*. [15] reported that several transgenic *Arabidopsis* plants that expressed chimeric repressors derived from the AtMYB102, ANAC047, HRS1, ZAT6, and AtERF5 transcription factors showed elevated salt stress tolerance [14].

In this study, we showed that six independent chimeric repressors derived from ADA2b, a transcription factor with Myb/SANT-like DNA-binding domain (Msantd), Dwarf and Delayed Flowering 1 (DDF1), dehydration responsive element binding protein 26 (DREB26), GL1 enhancer binding protein (AtGeBP), and homeobox protein 23 (ATHB23) from *A. thaliana* confer tolerance to salt and osmotic stress in *A. thaliana*.

## 2. Results and Discussion

### 2.1. Selection of Salt Tolerant CRES-T Lines

**Figure A1:**
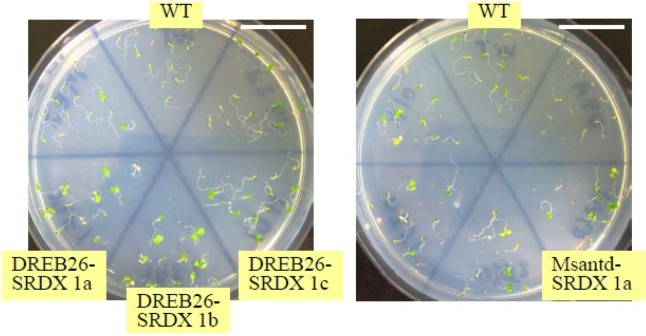
Screening of salt tolerant CRES-T lines. Seeds of CRES-T lines and WT were germinated on 1/2 MS medium supplemented with 175 mM NaCl for 14 days. Representative photographs, in which three DREB26-SRDX and Msantd-SRDX lines were used, are shown. Scale = 2 cm.

**Figure A2:**
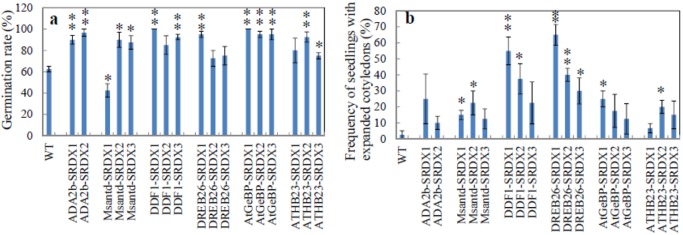
Salt tolerance of CRES-T lines. (**a**) Germination rate of CRES-T lines and WT was determined at 4 days after sowing on 1/2 MS medium supplemented with 175 mM NaCl; (**b**) Frequency of seedlings with expanded cotyledons was determined at 5 days after sowing on 1/2 MS medium supplemented with 175 mM NaCl. Ten seeds were used for each quadruple replication test. * and **: Significantly different from WT under non-stress condition at *p* < 0.05 and 0.01, respectively. ADA2b-SRDX1, Msantd-SRDX1, DDF1-SRDX1, DREB26-SRDX1, AtGeBPSRDX1, and ATHB23-SRDX2 were used for further experiments.

The primary screen of the transgenic *Arabidopsis* lines expressing the independent chimeric repressors from *A. thaliana* (CRES-T lines), yielded 234 putative salt-tolerant seedlings. These plants were rescued and grown in soil to maturity, and T2 seeds were harvested from individual plants. The T2 seeds from the individual putative salt-tolerant CRES-T lines were subjected to a secondary screen. Among them, CRES-T lines for ADA2b (AT4G16420), Msantd (AT4G31270), DDF1 (AT1G21610), DREB26 (AT1G21910), AtGeBP (AT1G11510), and ATHB23 (AT1G26960) listed in Table 1 showed higher germination rate and/or earlier development of cotyledons than WT plants on medium, supplemented with 175 mM NaCl (Appendix Figure A1). We prepared at least five independent lines for each selected chimeric repressor gene and confirmed their elevated salt tolerance at T2 and T3 lines that were homozygous for the transgene. Selected two or three lines from each construct showed better performance than WT when their germination rate and/or frequency of seedlings with expanded cotyledons were evaluated on 1/2 MS medium supplemented with 175 mM NaCl (Appendix Figure A2).

**Table 1:** List of Chimeric REpressor gene-Silencing Technology (CRES-T) lines with elevated salt tolerance.

CRES-T lines	Transcription factors converted to chimeric repressors			
	Locus	Description	Alias	Family
ADA2b-SRDX	AT4G16420	DNA binding/transcription coactivator/transcription factor	PRZ1 ADA2B	MYB
Msantd-SRDX	AT4G31270	A transcription factor with Myb/SANT-like DNA-binding domain	Msantd	Trihelix
DDF1-SRDX	AT1G21610	Wound-responsive family protein	DDF1	Wound-responsive
DREB26-SRDX	AT1G21910	AP2 domain-containing transcription factor family protein	DREB26	AP2-EREBP
AtGeBP-SRDX	AT1G11510	DNA-binding storekeeper protein-related	AtGeBP	GeBP
ATHB23-SRDX	AT1G26960	DNA binding/transcription factor	ATHB23	HB

### 2.2. Germination of CRES-T Lines on Medium Supplemented with Different Concentrations of NaCl and Mannitol

#### 2.2.1. Germination Rate

**Figure 1:**
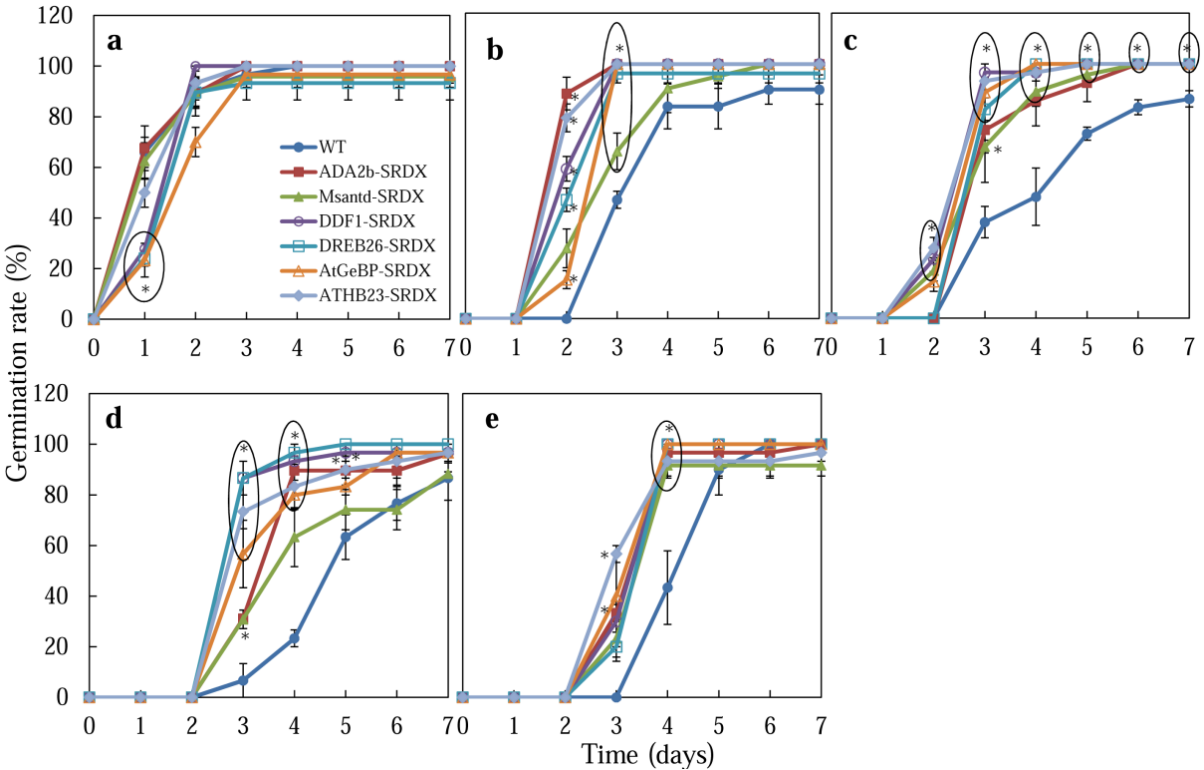
Germination rates of Wild type (WT) and CRES-T lines (ADA2b-SRDX, Msantd-SRDX, DDF1-SRDX, DREB26-SRDX, AtGeBP-SRDX, and ATHB23-SRDX) were monitored on medium supplemented with 0 (**a**), 150 (**b**), 175 (**c**), and 200 (**d**) mM NaCl and 400 mM mannitol (**e**). A seed was regarded as germinated when the radicle protruded through the seed coat. Similar experiments were performed three times with similar results. Error bars indicate the standard error of the mean. Ten seeds were used for each triplet replication test. * Significantly different from WT under non-stress condition at *p* < 0.05.

**Figure 2:**
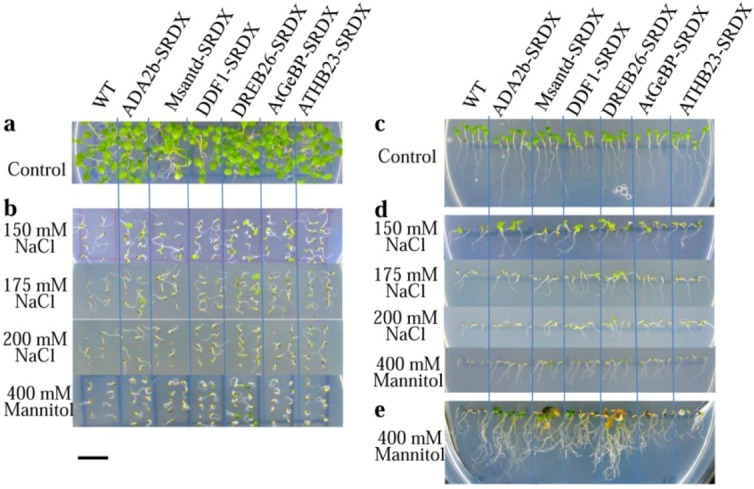
Seedlings of CRES-T and WT plants on medium supplemented with different concentrations of NaCl and mannitol. (**a**) Photograph of plants on control (non-stress) medium at 4 days after sowing; (**b**) Seedlings on medium supplemented with 150, 175, and 200 mM NaCl and 400 mM mannitol at 7 days after sowing; (**c**) Root development of CRES-T and WT plants on control (non-stress) medium at 4 days after sowing; (**d**) Comparison of root development between CRES-T lines and WT plants on medium supplemented with 150, 175, and 200 mM NaCl and 400 mM mannitol at 7 days after sowing; (**e**) Appearances of CRES-T and WT seedlings on medium supplemented with 400 mM mannitol at 4 weeks after sowing. Scale = 1 cm.

**Figure A3:**
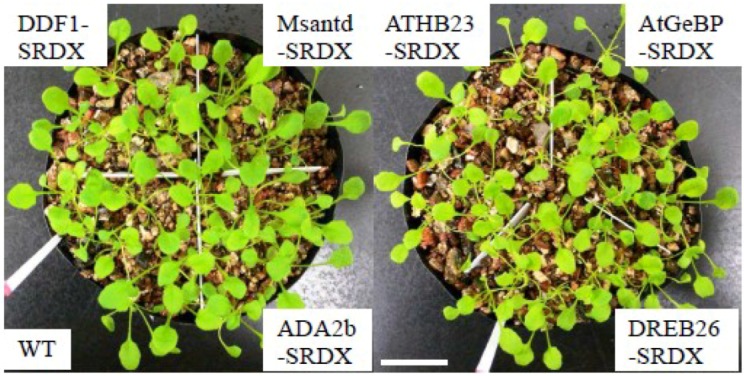
Growth of CRES-T lines and WT plants in pots with vermiculite under non-stressed conditions at 20 days after sowing. Scale = 2 cm.

Seeds of CRES-T lines and WT plants were grown on medium supplemented with 150, 175, and 200 mM NaCl or 400 mM mannitol, and the germination rates were analyzed (Figure 1 and Figure 2). We found that the CRES-T lines that expressed the chimeric repressor for ADA2b, Msantd, DDF1, DREB26, AtGeBP, and ATHB23 (ADA2b-SRDX, Msantd-SRDX, DDF1-SRDX, DREB26-SRDX, AtGeBP-SRDX, and ATHB23-SRDX, respectively) exhibited significantly higher tolerance to salt and osmotic stresses than WT plants. On medium without NaCl, almost 100% WT seeds and those from CRES-T lines germinated within 3 days after sowing (DAS) (Figure 1a). Under 150, 175, and 200 mM NaCl, all six CRES-T lines germinated earlier or exhibited significantly higher germination rates than WT plants (Figure 1b–d). After 4 weeks of incubation under 150 mM NaCl, seedlings of ADA2b-SRDX, Msantd-SRDX, DDF1-SRDX, DREB26-SRDX, and AtGeBP-SRDX lines retained green leaves, while ATHB23-SRDX and WT plants were predisposed to dying (data not shown). Under 400 mM mannitol, all six CRES-T lines germinated earlier (by 3 DAS) than WT (at 4 DAS), although the germination rates of both WT and all six CRES-T lines reached almost 100% by 5 DAS (Figure 1e). These experiments were repeated at least three times, and similar results were observed, although survival rates slightly differed among independent experiments. These six CRES-T lines grew well on soil when transferred from 1/2 MS plates and developed normally without any morphological defects (Appendix Figure A3).

**Figure 3:**
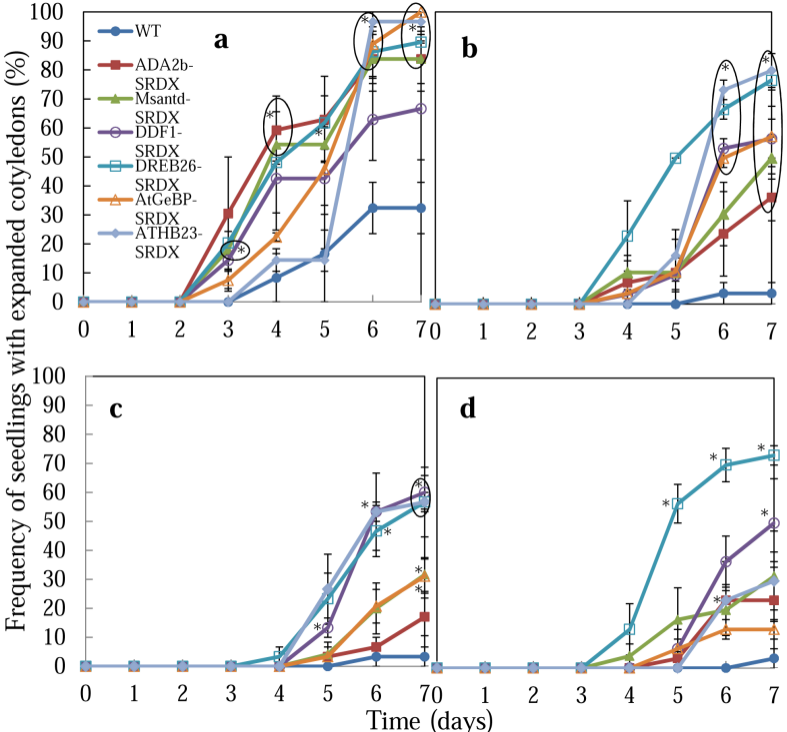
Effect of salt and osmotic stress on seedling growth of CRES-T plants. Seeds of WT and CRES-T lines (ADA2b-SRDX, Msantd-SRDX, DDF1-SRDX, DREB26-SRDX, AtGeBP-SRDX, and ATHB23-SRDX) were germinated on medium supplemented with 150 (**a**), 175 (**b**), and 200 (**c**) mM NaCl and 400 mM mannitol (**d**), and the numbers of seedlings with visible cotyledons were scored. Similar experiments were performed three times with similar results. Error bars indicate the standard error of the mean. Ten seeds were used for each triplet replication test. Scale = 1 cm.

#### 2.2.2. Rate of Cotyledon Appearance among Seedlings

Germination of CRES-T lines and WT plants was also evaluated by the rate of emergence of seedlings with cotyledons under stress conditions (Figure 2 and Figure 3). On medium without NaCl, no difference was observed among WT and CRES-T lines (Figure 2a). Under the 150 mM NaCl condition, appearance of green cotyledons was observed earlier in CRES-T lines, except for ATHB23-SRDX, than in WT (Figure 2b and Figure 3a). Under 175 and 200 mM NaCl, cotyledons appeared significantly earlier in all CRES-T lines than in WT (Figure 2b, Figure 3b and Figure 3c). Under 175 mM NaCl, some ADA2b-SRDX and DREB26-SRDX seedlings developed green expanded cotyledons; however, cotyledons of the other CRES-T lines and WT plants remained small and pale yellow at 7 DAS (Figure 2b and Figure 3b), indicating that ADA2b-SRDX and DREB26-SRDX possessed elevated salt tolerance compared with the other CRES-T lines. Under 400 mM mannitol, all CRES-T lines (at 4 to 6 DAS) showed earlier appearance of cotyledons than WT plants (at 7 DAS), and DREB26-SRDX showed the highest rate of seedlings with cotyledons (approximately 70%) at 7 DAS (Figure 3d). After 4 weeks of incubation under 400 mM mannitol, seedlings of ADA2b-SRDX, Msantd-SRDX, DDF1-SRDX, and DREB26-SRDX lines often developed and retained green leaves, while most seedlings of AtGeBP-SRDX, ATHB23-SRDX, and WT died (Figure 2e). In particular, most ADA2b-SRDX seedlings retained small but green leaves (Figure 2e). 

#### 2.2.3. Root Growth

**Figure 4:**
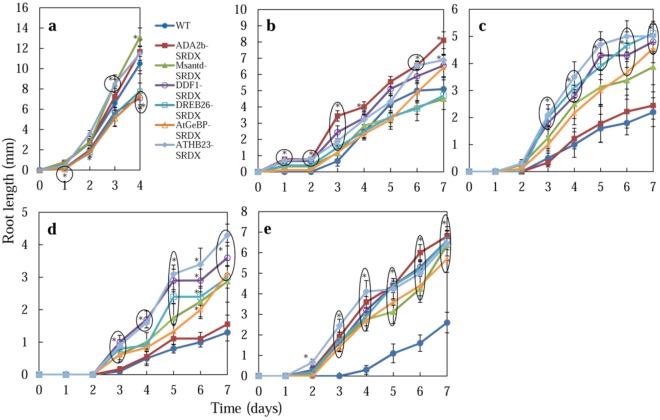
Effect of salt and osmotic stress on root growth of CRES-T plants. Seeds of WT and CRES-T lines (ADA2b-SRDX, Msantd-SRDX, DDF1-SRDX, DREB26-SRDX, AtGeBP-SRDX, and ATHB23-SRDX) were germinated on medium supplemented with 0 (**a**), 150 (**b**), 175 (**c**), and 200 (**d**) mM NaCl and 400 mM mannitol (**e**), and the root length of seedlings was scored. Similar experiments were performed three times with similar results. Error bars indicate the standard error of the mean. * Significantly different from WT under non-stress condition at *p* < 0.05. n = 10.

The root length of CRES-T lines and WT plants was measured during their germination on medium containing salt or mannitol (Figure 2 and Figure 4). On medium without salt, ADA2b-SRDX, Msantd-SRDX, and DDF1-SRDX lines showed root growth comparable with WT plants by 4 DAS; DREB26-SRDX, AtGeBP-SRDX, and ATHB23-SRDX lines developed shorter roots than WT plants (Figure 2c and Figure 4a). Under 150 mM NaCl, ADA2b-SRDX, DDF1-SRDX, DREB26-SRDX, and ATHB23-SRDX showed significantly better root growth than WT plants by 7 DAS; Msantd-SRDX and DREB26-SRDX showed root growth comparable with that of WT plants (Figure 2d and Figure 4b). Under 175 and 200 mM NaCl, all CRES-T lines except ADA2b-SRDX showed better root growth than WT plants at 3 DAS and later (Figure 2d, Figure 4c and Figure 4d). Under 400 mM mannitol, all CRES-T lines showed significantly better root growth than WT plants at 3 DAS and later (Figure 2d and Figure 4e). ADA2b-SRDX showed relatively better root growth after 2 weeks and later under osmotic stress conditions (Figure 2e).

#### 2.2.4. Possible Roles of Six Transcription Factors in Salt and Osmotic Stress Tolerance

Thus, all six CRES-T lines showed elevated salt and osmotic stress tolerance compared with WT plants. Among these, DREB26-SRDX showed higher tolerance than other lines at the germination stage to both salt and osmotic stress conditions, and ADA2b-SRDX showed higher tolerance at germination and during subsequent vegetative growth stages to osmotic stress conditions. No visible growth retardation was observed in the CRES-T lines under normal conditions (Appendix Figure A3).

ADA2b-SRDX showed the highest osmotic stress tolerance among the CRES-T lines. In *Arabidopsis*, two genes, *ADA2a* and *ADA2b*, encode proteins that resemble yeast ADA2 [16]. The transcriptional coactivator ADA2b is a component of GCN5-containing complexes controlling histone acetylation [17]. The mechanism by which ADA2b-SRDX gained elevated germination rate and root growth under salt stress (Figure 1, Figure 2, Figure 3 and Figure 4) requires further investigation. *Msantd* encodes a transcription factor with a Myb/SANT-like DNA-binding domain. The function of this transcription factor is unknown, but our data suggest that it must be involved in regulation of salt and osmotic stress tolerance. *DDF1* encodes an AP2 transcription factor of the *DREB1/CBF* subfamily. *ddf1 is* a gibberellin (GA)-deficient mutant and the *ddf1* mutation up-regulates a stress-related gene, *RD29A* [18]. *DDF1* mRNA is strongly induced by high-salinity stress. Moreover, transgenic plants overexpressing *DDF1* showed increased tolerance to high-salinity stress and caused dwarfism mainly by the reduction of the levels of bioactive gibberellin (GA) in transgenic *Arabidopsis* [19]. Under salinity stress, *Arabidopsis* plants actively reduce endogenous GA levels via the induction of GA2-oxidase, resulting in repressed growth for stress adaptation [19]. Mitigated dwarfism observed in DDF1-SRDX under salinity stress might be caused by maintaining endogenous GA levels by repressing the function of *DDF1*. This release from growth retardation under salt stress appears not to be a stress adaptation, rather a waste of resource under unfavorable condition. Thus, the DDF1-SRDX line that showed improved growth at the germination stage could not survive prolonged salt stress. DREB26 is a transcription factor belonging to the APETALA2/ethylene-responsive element (AP2-ERE) binding proteins family that includes DREB1A. Expression of *DREB26* responded to chitin [20], jasmonic acid, and ethylene treatments [21]. Transgenic *Arabidopsis* plants overexpressing *DREB26* exhibited abnormal morphology with tiny leaves, few or no secondary branches, and deformed flowers [21]. As DREB26-SRDX showed enhanced salt and osmotic stress tolerance, DREB26 may play an important role as a transcription factor regulating the stress tolerance pathway in *Arabidopsis*. *AtGeBP* encodes a DNA-binding storekeeper protein-related transcriptional regulator. Although the function of AtGeBP is unknown, the protein shows similarities to GL1 enhancer binding protein (GeBP) that binds and regulates the *GLABROUS* (*GL1*) gene, which is required for trichome initiation [22]. *ATHB23* encodes a homeodomain leucine zipper class I (HD-Zip I) protein and was predominantly expressed in siliques, rosette leaves, and inflorescences [23,24]. The roles of the GeBP homolog and the HD-Zip I in stress tolerance require further investigation. 

### 2.3. Salt Tolerance Test in Rosette-Stage Plants

**Figure A4:**
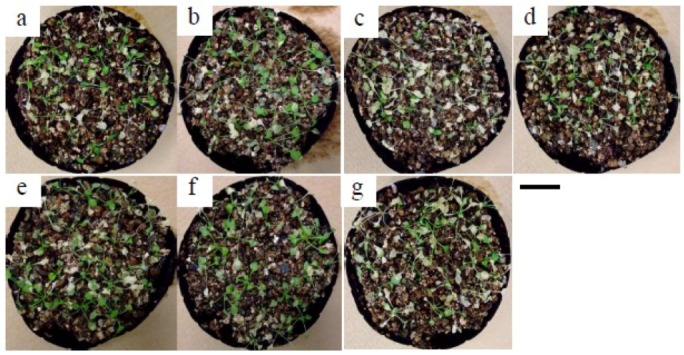
Salt tolerance test in rosette-stage plants. Three-week old CRES-T lines and WT plants planted in pots with vermiculite were subjected to 400 mM NaCl solutions for 7 days, and followed by treatment with salt-free water for a further 7 days. (**a**) WT, (**b**) ADA2b-SRDX, (**c**) Msantd-SRDX, (**d**) DDF1-SRDX, (**e**) DREB26-SRDX, (**f**) AtGeBP-SRDX, and (**g**) ATHB23-SRDX. Scale = 2 cm.

We evaluated salt tolerance of the CRES-T lines at the rosette (vegetative and flowering) stage by comparing survival rates of the CRES-T lines and WT plants in the presence of NaCl. When 3-week-old CRES-T lines and WT plants, which had been grown on vermiculite, were treated with 400 mM NaCl solution for 14 days, damage and the survival rates of the six CRES-T lines and WT plants were similar, or the CRES-T lines were more sensitive to salt stress (data not shown). When 3-week-old plants were treated with 400 mM NaCl solutions for 7 days, followed by treatment with salt-free water for a further 7 days, both CRES-T lines and WT plants showed similar damage to leaves and no difference was observed in their survival rate (Appendix Figure A4). These results suggest that salt tolerance of the CRES-T lines is comparable to that of WT plants at the rosette (vegetative and flowering) stage.

### 2.4. Expression of Transcription Factor, Chimeric Repressor and Stress-Related Genes in CRES-T Lines

**Figure A5:**
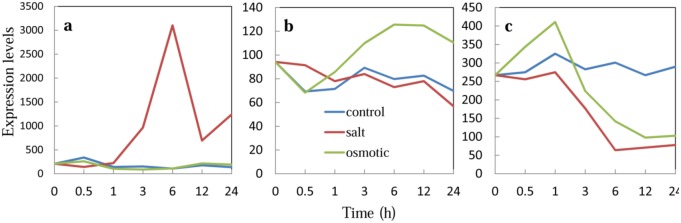
Expression of *Msantd*, *DREB26*, and *ATHB23* in WT plants. Absolute expression levels of each transcription factor gene in WT plants under salt (150 mM NaCl) and osmotic (300 mM mannitol) stress conditions were extracted from the AtGenExpress Visualization Tool and plotted as a graph. (**a**) Expression of *DREB26* in WT roots; (**b**) expression of *Msantd* in WT roots; and (**c**) expression of *ATHB23* in WT roots.

To analyze the gene expression profiles of the six transcription factor genes in WT plants under salt and osmotic stress conditions, we extracted gene expression data for the transcription factors under abiotic stress conditions from the AtGenExpress Visualization Tool [25]. Expression of *DREB26* in WT roots was up-regulated approximately 12-fold at 6 h after salt stress (150 mM NaCl) treatment but remained unchanged after osmotic stress (300 mM mannitol) treatment (Appendix Figure A5a), suggesting a role of DREB26 in salt tolerance in *Arabidopsis* roots. Expression levels in shoots were down-regulated to less than one tenth by both salt and osmotic stress treatment; however, expression in WT plants was also down-regulated to a similar level (data not shown), suggesting that DREB26 may not function in salt tolerance in shoots. *Msantd* was up-regulated 1.5-fold in mannitol-treated roots (Appendix Figure A5b), but not in shoots or in salt-treated shoots and roots, suggesting possible roles of *Msantd* in salt tolerance in roots. Expression of *ATHB23* in roots was down-regulated to less than 37% of that of untreated plants by both salt and osmotic stress treatment (Appendix Figure A5c). Changes in expression levels of the other transcription factor genes, *ADA2b*, *DDF1*, *AtGeBP*, and *ATHB23*, were relatively stable and remained between 60% and 130% of that of control plants after stress treatments (data not shown). 

**Figure 5:**
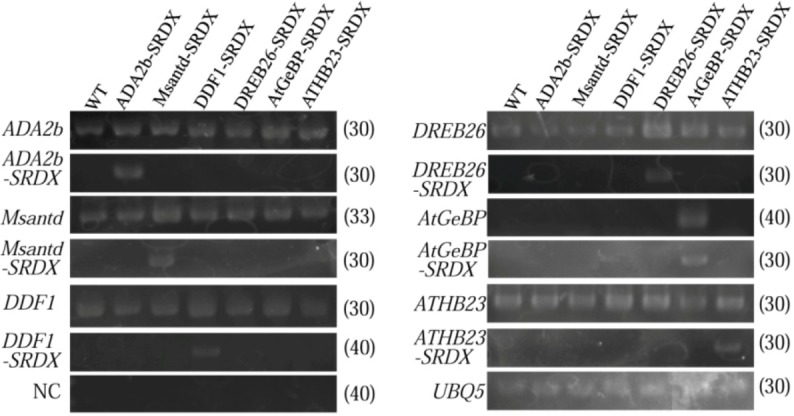
Expression levels of genes for six transcription factor and their chimeric repressor in CRES-T lines and WT plants was detected by RT-PCR. *UBQ5* was used as an internal control. NC; negative control; PCR using mix primers to detect transcripts for all the six transcription factors and RNAs without RT reaction as templates. The figures in parentheses are the PCR cycle numbers to detect the products.

To confirm the chimeric repressor genes are actually expressed in CRES-T lines, we performed RT-PCR analysis of CRES-T lines and WT plants using specific primers for each transcription factor gene and SRDX domain (Figure 5). Each of the chimeric repressor genes was expressed specifically in the corresponding CRES-T line. 

**Figure 6:**
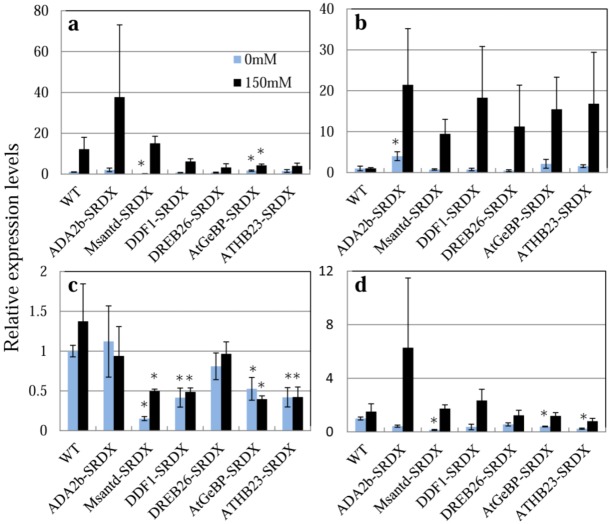
Expression of four salt tolerance-related genes, (**a**) *RD29A* (AT5G52310.1); (**b**) *RD22* (AT5G25610.1); (**c**) *DREB1A* (AT4G25480.1); and (**d**) *P5CS* (AT2G39800.1) in CRES-T lines and WT plants, was monitored by quantitative real-time RT-PCR. Ten-day-old seedlings of the CRES-T lines and WT on 1/2 MS plates were transplanted onto plates containing 1/2 MS medium supplemented with or without 150 mM NaCl. The seedlings were harvested at 24 h after transplantation followed by RNA extraction and quantitative real-time RT-PCR. Expression levels relative to WT under non-stress conditions (1.0) are shown. Error bars indicate the standard error of the mean. * Significantly different from WT under non-stress condition at *p* < 0.05. n = 3.

We monitored the expression of four stress-related genes, *RD29A*, *RD22*, *DREB1A*, and *P5CS*, in the CRES-T lines and WT plants after transferring to 1/2 MS medium containing 0 or 150 mM NaCl (Figure 6). It has been reported that *RD29A*, *RD22*, and *DREB1A* are involved in signal transduction in response to drought and salt stress [6,26,27,28]. P5CS encoding delta 1-pyrroline-5-carboxylate synthase controls the rate-limiting step of glutamate-derived biosynthesis of proline, which is associated with osmoregulation [29,30]. *RD29A* tended to be up-regulated in all six CRES-T lines and WT plants by 24 h of salt treatment; however, the magnitude of induction was 3 times greater in ADA2b-SRDX but smaller in DDF1-SRDX, DREB26-SRDX, AtGeBP-SRDX, and ATHB23-SRDX compared to WT plants (Figure 6a). Activation of the *RD29A*-containing pathway may be involved in the enhanced salt tolerance of ADA2b-SRDX. Expression of *RD29A* in Msantd-SRDX under non-stress condition and AtGeBP-SRDX under both stress and non-stress conditions was significantly lower than that of WT. Expression of *RD22* in ADA2b-SRDX was significantly higher than that of WT plants, even under non-stress condition (Figure 6b). Expression of *RD22* was reported to be up-regulated a few hours after salt treatment and returned to normal levels in WT plants at 24 h after salt treatment [31]; however, expression in the six CRES-T lines tended to remained at levels that were 10- to 20-fold higher than those in WT at 24 h after salt treatment (Figure 6b). Thus, activation of the *RD22*-containing pathway may be involved in the enhanced salt tolerance of the six CRES-T lines. Expression of *DREB1A* in ADA2b-SRDX and DREB26-SRDX was similar to that in WT under both non-stress and salt-stress conditions (Figure 6c). However, the expression of *DREB1A* in the other CRES-T lines was significantly repressed compared with WT plants and expression was not induced by salt treatment for 24 h (Figure 6c). Expression of P5CS in ADA2b-SRDX tended to be up-regulated under salt stress, suggesting that proline biosynthesis may be involved in the enhanced salt and osmotic stress tolerance of ADA2b-SRDX. Thus, six independent chimeric repressors derived from ADA2b, Msantd, DDF1, DREB26, AtGeBP, and ATHB23 from *A. thaliana* confer tolerance to salt and osmotic stress in *Arabidopsis*. In the CRES-T lines, expression of stress-protective genes was induced, possibly upon suppression of a transcription factor by the activity of a chimeric repressor. It was hypothesized that negative regulators act to fine-tune the suppression of bursts of expression of stress-response genes because overexpression of such genes may have adverse effects on plant development [32,33,34]. Our results, in addition to the results by Mito *et al*. [15], support this hypothesis. 

## 3. Experimental

### 3.1. Generation and Selection of CRES-T Lines

CRES-T lines for independent 1600 transcription factors from *A. thaliana* were produced according to Mito *et al*. [15] (Ohme-Takagi *et al*. unpublished data, will be published elsewhere) using the binary vector pBCKH, which contain CaMV35S promoter for chimeric gene expression and the *hygromycin phosphotransferase* gene for selection of transformants. T2 seeds of each CRES-T lines and WT plants (Col-0 ecotype) were sown on 1/2 MS medium supplemented with 1% sucrose, 175 mM NaCl, and 0.8% agar. Plates were incubated in growth chambers at 23 °C under a 16 h light/8 h dark cycle with a photon flux density of 350 μmol photons m^−2^ s^−1^. After 14 days of incubation, growth of CRES-T seedlings was visually compared with that of WT plants.

### 3.2. Stress Tolerance Assay

T2 or T3 seeds from CRES-T lines and WT plants were sown on 1/2 MS medium with 1% sucrose, 0.8% agar, and different concentrations of NaCl or mannitol, and the plates were placed at 4 °C for 3 days and then transferred to normal growth conditions. For 7 days of incubation, the percentage of germinated seeds and seedlings with developed cotyledons and the root length of germinated seedlings were determined. A seed was regarded as germinated when the radicle protruded through the seed coat. For assays in rosette plants, 3-week-old CRES-T lines and WT plants that were grown in vermiculite were treated with 400 mM NaCl solutions for 2 weeks or were treated for 1 week followed by treatment with salt-free water for a further 1 week. NaCl solution was replaced with fresh solution twice per week.

### 3.3. Extraction of Gene Expression Profiles of Transcription Factors

Gene expression profiles of the six transcription factors in WT plants under salt and osmotic stress conditions were extracted using the AtGenExpress Visualization Tool [25]. 

### 3.4. Quantitative Real-Time RT-PCR and RT-PCR

The expression of five salt tolerance-related genes, *RD29A* (AT5G52310.1), *RD22* (AT5G25610.1), *DREB1A* (AT4G25480.1), and *P5CS* (AT2G39800.1) was monitored by quantitative real-time RT-PCR following the methods detailed in our previous report [35]. The primers used are listed in Table 2. Ten-day-old seedlings of CRES-T lines and WT on 1/2MS plates were transplanted onto 1/2 MS-medium plates supplemented with or without 150 mM NaCl, and the seedlings were then harvested at 24 h after transplantation. Total RNA was isolated from the seedlings of each of three biological replicates using the RNeasy Plant RNA extraction kit (Qiagen, Tokyo, Japan). Single strand cDNA was synthesized from 500 ng of total RNA using a QuantiTect Reverse Transcription Kit (Qiagen) according to the manufacturer’s instructions. A total of 1 µL of a 40-fold dilution of the reaction mixture was subsequently used for PCR. Real-time quantitative PCR was performed using a QuantiTect SYBR Green PCR Kit (Qiagen) with a LightCycler (Roche Diagnostics, Basel, Switzerland). The delta-delta Ct method was used to calculate the relative gene expression using *ubiquitin extension protein* (*UBQ5*, AT3G62250.1) as an internal control to normalize all data. The expression of chimeric repressor genes was detected by RT-PCR. Single strand cDNAs synthesized from RNAs from unstressed CRES-T lines and WT plants were used as templates. PCR was performed using ExTaq DNA polymerase (Takarabio, Ohtsu, Japan). The primers used are listed in Table 2.

**Table 2:** List of primers used.

Genes	primers	Sequences
	35S-F	5'-CGGATTCCCATTGCCCAGCTATCT-3'
	NOS-R	5'-TAATCATCGCAAGACCGGCAACAG-3'
*RD29A*	Forward	5'-AAGAGGAAGTGAAAGGAGGAGGAGTCACGC-3'
	Reverse	5'-CACCACCAAACCAGCCAGA-3'
*RD22*	Forward	5'-GTCTTCCTCTGATCTGTCTTCTTGG-3'
	Reverse	5'-TGGGAATGGGAGTGTTTGG-3'
*DREB1A*	Forward	5'-atatgcacgatgaggcgatg-3'
	Reverse	5'-tcatcatcgccgtcgacttc-3'
*P5CS*	Forward	5'-AGTCGGGGTCGAAGGATTAC-3'
	Reverse	5'-gcttggatgggaatgtcctg-3'
*UBQ5*	Forward	5'-tgtgaaggcgaagatccaag-3'
	Reverse	5'-gagacggaggacgagatgaag-3'
*ADA2b*	Forward	5'-cggctcaaagatctcaaggaagc-3'
	Reverse	5'-ggctatatgcatccgacttcttcg-3'
*Msantd*	Forward	5'-gccatcaatgcggttgtgatgatcc-3'
	Reverse	5'-cactcagacaaccgatgagctcg-3'
*DDF1*	Forward	5'-ggtaccaagaacagactgtactgc-3'
	Reverse	5'-tgaggagctgggattacacgtgtc-3'
*DREB26*	Forward	5'-caacagctgaagcagctgctagagc-3'
	Reverse	5'-gcggaatgtcagcatcttcgtagaag-3'
*AtGeBP*	Forward	5'-cgtcaaacctcatgatcgcaaggc-3'
	Reverse	5'-agcctgcatcgctttccactgctcg-3'
*ATHB23*	Forward	5'-agctagctcgtgccttgggattgc-3'
	Reverse	5'-tgctggtcaagccatggccagaac-3'
*SRDX*	SRDX-R	5'-ttaagcgaaacccaaacggagttctag-3'

### 3.5. Statistical Analysis

Statistical significance was determined using an unpaired Student’s *t*-test at the 0.05 or 0.01 probability level.

## 4. Conclusions

Our data revealed that the transcription factors ADA2b, Msantd, DDF1, DREB26, AtGeBP, and ATHB23 play roles in salt and osmotic stress tolerance in *A. thaliana*. Mito *et al*. [15] showed that expression of a chimeric repressor provides an effective strategy for enhancing tolerance of plants to abiotic stress via a similar experiment using CRES-T lines. Our results provide additional examples of CRES-T lines with enhanced stress tolerance and further proof that CRES-T is a powerful tool for revealing the function of transcription factors. Analyzing the mechanisms in which these six transcription factors could play roles in salt and osmotic tolerance will provide novel insight into the study of abiotic stress tolerance in plants.
